# The Physicochemical Compatibility of Sildenafil Injection with Parenteral Medications Used in Neonatal Intensive Care Settings

**DOI:** 10.3390/pharmaceutics16030419

**Published:** 2024-03-18

**Authors:** D. Thisuri N. De Silva, Tobias Strunk, Michael Petrovski, Madhu Page-Sharp, Brioni R. Moore, Kevin T. Batty

**Affiliations:** 1 Curtin Medical School, Curtin University, Bentley 6102, Australia; d.desilva2@postgrad.curtin.edu.au (D.T.N.D.S.); m.page-sharp@curtin.edu.au (M.P.-S.); brioni.moore@curtin.edu.au (B.R.M.); 2 Medical School, The University of Western Australia, Crawley 6009, Australia; tobias.strunk@health.wa.gov.au; 3 Wesfarmers Centre for Vaccines and Infectious Diseases, Telethon Kids Institute, Nedlands 6009, Australia; 4 Neonatal Directorate, King Edward Memorial Hospital, Child and Adolescent Health Service, Subiaco 6008, Australia; 5 Pharmacy Department, King Edward Memorial Hospital, Women and Newborn Health Service, Subiaco 6008, Australia; michael.petrovski@health.wa.gov.au; 6 Curtin Health Innovation Research Institute, Curtin University, Bentley 6102, Australia

**Keywords:** sildenafil, physical compatibility, chemical compatibility, neonates, syringe filters

## Abstract

Sildenafil is used to treat pulmonary hypertension in neonatal intensive care unit (NICU) settings. As multiple intravenous (IV) medications are co-administered in NICU settings, we sought to investigate the physicochemical compatibility of sildenafil with a range of IV drugs. Sildenafil 600 mcg/mL or 60 mcg/mL was mixed 1:1 with the secondary drug solution to simulate Y-site co-administration procedures. Physical compatibility was evaluated by visual observation against a black and white background and under polarized light for two hours for changes in colour, precipitation, haze and evolution of gas. Chemical compatibility was determined from sildenafil concentrations, using a validated, stability-indicating high-performance liquid chromatography assay. Sildenafil 600 mcg/mL was physicochemically compatible with 29 of the 45 drugs tested at ‘high-end’ clinical concentrations and physically incompatible with 16 drugs and six ‘2-in-1’ parenteral nutrition solutions. Sildenafil 600 mcg/mL was compatible with lower, clinically relevant concentrations of calcium gluconate, heparin and hydrocortisone. Aciclovir, amoxicillin, ampicillin, ibuprofen lysine, indometacin, phenobarbitone and rifampicin were incompatible with sildenafil 600 mcg/mL, however these IV medications were compatible with sildenafil 60 mcg/mL. Sildenafil 600 mcg/mL and 60 mcg/mL were incompatible with amphotericin, flucloxacillin, furosemide, ibuprofen, meropenem and sodium bicarbonate. Sildenafil compatibility with commonly used syringe filters was also investigated. Sildenafil solution was compatible with nylon syringe filters, however, absorption/adsorption loss occurred with polyethersulfone and cellulose ester filters.

## 1. Introduction

Sildenafil is a phosphodiesterase type 5 inhibitor used in the second-line management of persistent pulmonary hypertension (PPHN) of the newborn, with proven reduction in mortality and a favourable adverse effect profile [1]. Conventional first-line treatment for PPHN is inhaled nitric oxide, however, as this is an expensive treatment modality, it is not commonly used in all countries. In addition, up to 50% of infants with PPHN may not respond to nitric oxide, therefore sildenafil has become a well-established second line therapy [2]. The conventional treatment regimen of intravenous (IV) sildenafil for PPHN is a loading dose of 0.4 mg/kg administered over three hours, followed by a continuous infusion of 1.6 mg/kg/day for up to seven days, with the sildenafil concentration typically in the order of 400–800 mcg/mL in glucose 5% *w*/*v* (D5W) injection [1]. In preterm infants, a lower loading dose of 0.1 mg/kg administered over 45 min and continuous infusion of 0.5 to 1.2 mg/kg/day is recommended, using sildenafil concentrations in the order of 60–100 mcg/mL in D5W injection [3].

In neonatal intensive care unit (NICU) settings, infants often require several concurrent IV medications, which may be at high concentrations due to fluid restrictions. Multiple IV access sites for these medications pose a threat of pain, risk of infection and thromboembolism to the patients [4]. Due to the limited vascular access in these patients, IV drug administration via a “Y-site” arrangement with three-way connectors is commonly used to infuse multiple drugs simultaneously [5]. Combined with low infusion flow rates and high drug concentrations, one risk with Y-site administration of IV drug combinations is physical and/or chemical drug incompatibilities in the IV apparatus [6]. Physical incompatibility can present as visible precipitates, haze, colour change or gas formation. Infusion of particulate matter of adequate size (i.e., larger than the 4–9 µm capillary diameter) into the vasculature of neonates can cause serious adverse embolic events and may be fatal [7]. Furthermore, chemical incompatibility may lead to sub-optimal clinical outcomes or adverse effects if toxic compounds are formed. Therefore, physicochemical compatibility should be carefully considered when IV drugs are co-administered via Y-sites, with due regard to concentrations and combinations that are applicable to the clinical setting, such as NICU [5].

Physicochemical compatibility of IV sildenafil with other drugs has been reported for pentoxifylline, epinephrine, norepinephrine, vasopressin, heparin, milrinone and dopamine. All drugs were found to be compatible with sildenafil at the concentrations tested, except heparin, which was compatible at 1 unit/mL and incompatible at 100 units/mL [8,9,10]. 

Against this background, we sought to investigate the physicochemical compatibility of sildenafil with a range of NICU drugs, at higher end clinically relevant concentrations, and with a selection of 2-in-1 parenteral nutrition (PN) solutions. 

## 2. Materials and Methods

Sildenafil (sildenafil citrate; C_22_H_30_N_6_O_4_S·C_6_H_8_O_7_; MW 666.7; certified reference material), was purchased from Sigma-Aldrich Chemicals, St. Louis, MO, USA. HPLC grade acetonitrile was from Fisher Scientific, Fair Lawn, NJ, USA. All other laboratory chemicals were of analytical grade. All parenteral medications and solutions were of clinical grade (see online Supplementary File for list of medications and manufacturers—Table S1). The composition of the 2-in-1 PN solutions is provided in Table S2 of the online Supplementary File.

### 2.1. High Performance Liquid Chromatography (HPLC) Assay

The Agilent 1200 series HPLC system comprised a binary pump with degasser, auto-sampler, thermostated column oven and a dual wavelength UV detector (Agilent Technology, Waldbronn, Germany). Chemstation software (vRev. B.03.01.SR1; Agilent Technology) was used to acquire and process data.

A reversed phase HPLC column (Kinetex, 5µm, C_18_; 100 × 4.6 mm; Phenomenex, Torrance, CA, USA) was maintained at 30 °C. The mobile phase was an isocratic mixture of 40% *v*/*v* acetonitrile and 60% *v*/*v* 50 mM potassium dihydrogen orthophosphate buffer (pH 6; HI 5221 pH Meter, Hanna Instruments, Woonsocket, RI, USA). The flow rate and UV detector were 1 mL/min and 240 nm, respectively. The injection volume was 5 µL, unless otherwise specified.

The stability-indicating HPLC method development was guided by previous studies [8,9,11] and validated in accordance with the International Council for Harmonization guidelines [12]. Sildenafil 600 mcg/mL was prepared by diluting sildenafil injection (Revatio; Viatris, Australia; Supplementary File—Table S1) with D5W and exposing it to forced degradation experiments with acidic, alkali and oxidative stress conditions.

Oxidative stress: Sildenafil 600 mcg/mL was mixed 1:1 with 20% *v*/*v* hydrogen peroxide (2 mL volume in 4 mL glass vials with impermeable caps, *n* = 3), and stored in a stability chamber at 45 °C (Fitoclima 600, Aralab, Rio de Mouro, Portugal). Samples (300 µL) were withdrawn at 0, 1, 2, 4 and 7 days and frozen (−80 °C) to arrest further degradation until assayed. At the time of assay, samples were thawed at ambient room temperature (22 °C), vortex mixed, diluted 1-in-50 with water, then analysed by HPLC as described above (injection volume 20 µL). 

Acid stress: Sildenafil 600 mcg/mL was mixed with 4 M hydrochloric acid (1:1 *v*/*v*; 2 mL in 4 mL glass vials with impermeable caps, *n* = 3), and stored at 45 °C. Samples (300 µL) were withdrawn at 0, 1, 2, 4 and 7 days, neutralised with 4 M sodium hydroxide solution and frozen (−80 °C). At the time of assay, samples were thawed, vortex mixed, diluted 1-in-50 with water and then analysed by HPLC as described above (injection volume 20 µL). 

Alkali stress: A similar process as described above for acid stress was followed, using 4 M sodium hydroxide solution and neutralisation with 4 M hydrochloric acid. 

Heat stress: Sildenafil 600 mcg/mL was mixed with water (1:1 *v*/*v*; 2 mL in 4 mL glass vials with impermeable caps, *n* = 3), and stored at 60 °C (PURA 4 water bath, Julabo GmbH, Seelbach, Germany). Samples (500 µL) were withdrawn at 0 and 3 days and frozen (−80 °C). At the time of assay, samples were thawed, vortex mixed and analysed by HPLC as described above (injection volume 5 µL).

Light stress: Sildenafil 600 mcg/mL was mixed with water (1:1 *v*/*v*; 2 mL in 4 mL glass vials with impermeable caps, *n* = 3) and exposed to light (laboratory fluorescent lighting 24/7 and normal daylight (indirect sunlight) for approximately 12 h per day) at room temperature (22 °C). Samples (500 µL) were withdrawn at 0 and 7 days and frozen (−80 °C). At the time of assay, samples were thawed, vortex mixed and analysed by HPLC as described above (injection volume 5 µL).

To establish linearity and range for the HPLC assay, a calibration curve was constructed using sildenafil solutions at concentrations of 3, 10, 30, 100, 300 and 800 mcg/mL (*n* = 3). Calibration curve and analyte concentration data were analysed using Microsoft Excel (Version 2309 Build 16.0.16827.20166). The limit of detection (LOD) and the lower limit of quantitation (LLOQ) were estimated using the formulae below, where σ is the residual standard deviation of a regression line and S is the slope of the calibration curve [12]. LLOQ was confirmed by precision data.

$$LOD=\frac{3.3\times \sigma }{S}$$

$$LLOQ=\frac{10\times \sigma }{S}$$

Accuracy and precision of the HPLC assay was evaluated at sildenafil concentrations of 600, 100, 10 and 2.9 (LLOQ) mcg/mL (*n* = 5) using the sildenafil reference standard and the commercial sildenafil injection diluted with D5W. The concentrations of the two series were compared (expressed as a fraction of the nominal concentration). Intra-assay and inter-assay precision were determined by calculating percentage relative standard deviation (%RSD) for the same sildenafil concentrations.

### 2.2. Preparation of Samples for Physical and Chemical Compatibility Testing

Sildenafil injection (800 mcg/mL) was diluted using D5W to achieve clinically relevant concentrations of 60 and 600 mcg/mL. The higher sildenafil concentration is consistent with a high-end dosage regimen for infants ≥37 weeks gestational age, and the lower sildenafil concentration is consistent with a low-end dosage regimen for pre-term infants <37 weeks gestational age [13]. Secondary test drugs and 2-in-1 PN solutions were prepared/diluted in accordance with the manufacturer’s instructions or standard local neonatal clinical protocols at King Edward Memorial Hospital. Drug concentrations were based on the recommendations for a patient weighing 2 kg (see Table 1 and Table 2 for secondary drug concentrations used in the present study). Medications that were originally contained in glass ampoules or required reconstitution were filtered immediately prior to mixing (33 mm × 0.22 µm Polyethersulfone (PES) membrane, Millex-GP, Merck Millipore Ltd., Carrigtwohill, Co., Cork, Ireland). 

A panel of 45 drugs and 6 PN solutions were selected and endorsed by local clinical experts (TS, MP). Five drugs were included in the study as positive (compatible: epinephrine, norepinephrine, milrinone and dopamine) or negative (incompatible: heparin 100 units/mL) controls, and the remaining forty drugs were previously untested against sildenafil. Epinephrine, norepinephrine, milrinone and dopamine were tested in the present study at different concentrations to previous reports [8,9].

Drug combinations (sildenafil and the test drug or PN solution) were mixed at a 1:1 (*v*/*v*) ratio to simulate Y-site administration, consistent with established methods [8,14,15,16,17]. Drug preparation, mixing and testing was carried out at room temperature (22 °C). 

The first stage of compatibility tests comprised a combination of sildenafil 600 mcg/mL and the secondary drug at clinically relevant high-end concentrations, consistent with the standard NICU protocols and expert advice. If incompatibility was detected, the drug combination was then tested using sildenafil 600 mcg/mL and the secondary drug at a low-end clinically relevant concentration, if applicable. The third and fourth stages of tests comprised sildenafil 60 mcg/mL and the secondary drug at high- and low-end concentrations, respectively, as applicable. The ‘up to four-way’ combination design optimised the scope for clinically relevant information on incompatible combinations.

Twelve 2 mL clear glass HPLC vials with impermeable screw cap lids were used for each binary combination of drugs/fluids and the respective control solutions. Sildenafil, secondary drug combinations and the control samples were prepared as described below:

- Set 1—Sildenafil injection solution (0.4 mL of 60 or 600 mcg/mL) and secondary test drug solution/fluid (0.4 mL); *n* = 4.
- Set 2—Sildenafil injection solution (0.4 mL of 60 or 600 mcg/mL) diluted with 0.4 mL of the diluent of the secondary test drug (*n* = 4) as the reference control solution for the purpose of visual comparison and HPLC assay of sildenafil concentration. The diluent was D5W for PN solutions.
- Set 3—The test drug solution/fluid (0.4 mL) was diluted with 0.4 mL of D5W (*n* = 4) for the purpose of visual comparison.

### 2.3. Physical Compatibility Testing

All vials were gently mixed and inspected with an unaided eye against a black and white background for any change in colour, haze or precipitation. The observations were carried out immediately after mixing, and 5, 15, 60 and 120 min. Samples were also observed under a polarized light viewer (Apollo I Liquid Viewer with a LED light source and 1.7× Magnifier, Adelphi Manufacturing Company Ltd., Haywards Heath, West Sussex, UK) for any visible precipitation or particulate matter. Physical compatibility was based on the visual appearance of the drug combination (set 1) in comparison to control solutions (set 2 and 3). Any inconclusive observation was confirmed by a second independent observer and all physical incompatibilities were photographed. If precipitation or particles were observed in the drug combination vials, an aliquot was examined under light microscopy (Leica MC190HD, 40× magnification, Leica Microsystems (Switzerland) Ltd., CH—9435, Heerbrugg, Switzerland).

### 2.4. Chemical Compatibility Testing

The HPLC assay was used to evaluate chemical compatibility if the combination was physically compatible. If any physical incompatibility was observed, such combinations were not chemically tested to avoid contamination of the HPLC system. At 2 h after mixing, the sildenafil concentration in the four vials of sildenafil plus test drug (set 1) was measured by HPLC and compared to the four sildenafil reference solution vials (set 2). The ratio of the mean peak areas was determined and the 95% CI of the ratio was calculated using the confidence limits from a two-sided *t*-test (α = 0.05; SigmaPlot V.15; Inpixon GmbH, Düsseldorf, Germany). Consistent with previous studies, incompatibilities of sildenafil:drug combinations were defined as a ratio of the mean peak area outside the range of 90–110% [8,9,18,19,20].

### 2.5. Evaluation of Absorption/Adsorption Loss of Sildenafil by Syringe Filters

The compatibility of sildenafil injection with conventional syringe filters has not previously been reported but is clinically relevant information and was required for subsequent tests in the present study. Six types of syringe filters and two inline filters composed of different filter membranes (cellulose esters, nylon, polyvinylidene fluoride, polyethersulfone and polypropylene; online Supplementary File Table S5) were tested to evaluate the absorption/adsorption loss of sildenafil during the process of filtration. 

Sildenafil 60 mcg/mL and 600 mcg/mL solutions were used for filter testing and the drug recovery in the filtrate was determined by HPLC assay. The peak area values obtained with and without filtration were compared and data were reported as percent recovery according to the following formula:

$$Recovery\;of\;sildenafil\;(\%)\;=\frac{sildenafil\;concentration(filtered;\;peak\;area\;of\;the\;chromatogram)}{sildenafil\;concentration\;of\;the\;unfiltered\;solution}\times 100$$

A pilot test was carried out using the eight filter types (online Supplementary File Table S5) and the two concentrations of sildenafil solution in D5W. Filtrate was collected as five separate, consecutive 1 mL portions of solution to examine the influence of the volume of filtrate on the drug recovery. Testing was carried out in triplicate and a new filter unit was used for each sample.

Based on the pilot study results, four filters were selected for further testing due to the recovery data and/or clinical relevance of the filters (see online Supplementary File Table S5): nylon (NY, 15 mm × 0.2 µm); Millex-GP (PES, polyethersulfone, 33 mm × 0.22 µm); Millex-GS (MCE, mixed cellulose esters, 33 mm × 0.22 µm); inline filter (polyethersulfone 25 mm × 0.2 µm). Sildenafil commercial injection solution (60 and 600 mcg/mL in D5W) was tested in a similar manner using a test volume of 4 mL (*n* = 3).

## 3. Results

### 3.1. HPLC Method Validation

The HPLC chromatograms revealed the sildenafil peak was well resolved from the solvent and degradation product peaks in all stress conditions tested. Sildenafil eluted at approximately 4.2 min whereas all degradation products eluted at less than 3 min (online Supplementary File Figures S1–S5). Oxidation of sildenafil resulted in the most extensive degradation profile, with a loss of 14.9% at the seventh day of exposure. Degradation products were detected at 1.5, 1.7 and 2.9 min. Alkali degradation of sildenafil was found to be 11.4% at the seventh day of exposure, with one degradation product detected at 0.9 min. Exposure of sildenafil to acid, heat and light showed no detectable degradation peaks, with post-exposure sildenafil drug concentrations of 98.5%, 103.6% and 99.2%, respectively. 

The assay was linear for sildenafil in aqueous solution (*n* = 3) within the concentration range 3–800 mcg/mL (r^2^ > 0.999) (online Supplementary File Figure S6). The LOD and LLOQ for sildenafil were 0.96 and 2.9 mcg/mL, respectively. The HPLC method was accurate and precise according to standard definitions [12], with accuracy being 100–105% for all samples and precision (%RSD) being <4.2% for inter- and intra-assay samples (online Supplementary File Table S3).

### 3.2. Sildenafil Compatibility

#### 3.2.1. Sildenafil 600 mcg/mL

Sildenafil 600 mcg/mL was physically and chemically compatible with 29 of the 45 drugs tested at high-end clinical concentrations in the present study: alprostadil, liposomal amphotericin, benzylpenicillin, caffeine (base), caffeine citrate, cefotaxime, ciprofloxacin, clonidine, cloxacillin, dexmedetomidine, dobutamine, dopamine, epinephrine, fentanyl, fluconazole, gentamicin, insulin, levetiracetam, linezolid, metronidazole, midazolam, milrinone, morphine hydrochloride, morphine sulfate, norepinephrine, paracetamol, piperacillin/tazobactam, vancomycin and vecuronium (Table 1). However, sildenafil 600 mcg/mL was physically incompatible with 16 drugs and all 6 of the 2-in-1 PN solutions, with precipitates and haziness occurring almost immediately (Table 1). In the first series of re-testing sildenafil 600 mcg/mL with secondary drugs at lower, clinically relevant concentrations, three of the combinations were found to be compatible (calcium gluconate 50 mg/mL; heparin 2 units/mL; hydrocortisone 1 mg/mL; Table 1). However, sildenafil 600 mcg/mL was incompatible with amoxicillin (100 mg/mL and 50 mg/mL), ampicillin (100 mg/mL and 50 mg/mL) and meropenem (50 mg/mL and 25 mg/mL) (Table 1). All physical incompatibilities were visible to the naked eye, except for the combination with calcium gluconate (100 mg/mL) which required polarized light for clear visualisation. Photographs of selected incompatible drug combinations and their corresponding photomicrographs can be found in the online Supplementary File (Figures S9–S12).

**Table 1:** Physicochemical compatibility of sildenafil 600 mcg/mL with secondary drugs and 2-in-1 parenteral nutrition solutions (see online Supplementary File Table S2 for details).

Secondary Drug	Test Concentration	Diluent	P/C *	SIL Ratio	95% CI of Ratio
Aciclovir	5 mg/mL	D5W	I ^a^	-	-
Alprostadil	20 mcg/mL	NS	C	99.9	99.4–100.4
Amoxicillin	100 mg/mL	WFI	I ^b^	-	-
Amoxicillin	50 mg/mL	WFI	I ^b^	-	-
Amphotericin (Fungizone)	100 mcg/mL	D5W	I ^b^	-	-
Amphotericin liposomal	2 mg/mL	D5W	C	99.9	99.0–100.8
Ampicillin	100 mg/mL	WFI	I ^b^	-	-
Ampicillin	50 mg/mL	WFI	I ^b^	-	-
Benzylpenicillin	100 mg/mL	WFI	C	101.2	99.7–102.7
Caffeine (base)	10 mg/mL	U	C	101.0	100.1–101.9
Caffeine citrate	20 mg/mL	U	C	100.4	99.6–101.2
Calcium gluconate	100 mg/mL	U	I ^c^	-	-
Calcium gluconate	50 mg/mL	NS	C	100.0	99.1–100.8
Cefotaxime	100 mg/mL	WFI	C	102.1	99.9–104.3
Ciprofloxacin	2 mg/mL	U	C	101.3	100.3–102.2
Clonidine	2 mcg/mL	NS	C	99.7	99.1–100.4
Cloxacillin	100 mg/mL	WFI	C	101.1	100.2–102.0
Dexmedetomidine	1 mcg/mL	NS	C	100.0	98.9–101.1
Dobutamine hydrochloride	7.2 mg/mL	NS	C	99.9	99.1–100.7
Dobutamine hydrochloride	7.2 mg/mL	D5W	C	100.4	99.5–101.3
Dopamine	7.2 mg/mL	NS	C	100.5	99.9–101.0
Dopamine	7.2 mg/mL	D5W	C	100.7	100.2–101.3
Epinephrine	64 mcg/mL	D5W	C	99.9	99.3–100.5
Fentanyl	50 mcg/mL	U	C	98.2	95.4–100.9
Flucloxacillin	50 mg/mL	D5W	I ^d^	-	-
Fluconazole	2 mg/mL	U	C	100.2	99.4–100.9
Furosemide	1 mg/mL	D5W	I ^b^	-	-
Furosemide	0.2 mg/mL	D5W	I ^b^	-	-
Gentamicin	10 mg/mL	WFI	C	101.9	101.3–102.5
Gentamicin	10 mg/mL	NS	C	102.2	100.4–104.0
Heparin	100 units/mL	NS	I ^d^	-	-
Heparin	2 units/mL	NS	C	99.1	98.3–100.0
Hydrocortisone	10 mg/mL	NS	I ^a^	-	-
Hydrocortisone	1 mg/mL	NS	C	99.7	93.2–106.1
Ibuprofen	5 mg/mL	NS	I ^e^	-	-
Ibuprofen lysine	4 mg/mL	NS	I ^e^	-	-
Indometacin	200 mcg/mL	NS	I ^e^	-	-
Insulin	0.2 units/mL	NS	C	100.5	98.6–102.4
Levetiracetam	5 mg/mL	NS	C	99.7	98.8–100.6
Linezolid	2 mg/mL	U	C	98.8	97.8–99.8
Meropenem	50 mg/mL	NS	I ^b^	-	-
Meropenem	25 mg/mL	NS	I ^b^	-	-
Metronidazole	5 mg/mL	U	C	99.2	98.3–100.1
Midazolam	1 mg/mL	U	C	100.3	99.9–100.8
Midazolam	120 mcg/mL	NS	C	99.9	98.6–101.2
Midazolam	120 mcg/mL	D5W	C	100.5	99.6–101.4
Midazolam	500 mcg/mL	NS	C	100.5	98.4–102.7
Milrinone	400 mcg/mL	D5W	C	100.5	99.5–101.4
Morphine hydrochloride	200 mcg/mL	D5W	C	100.4	99.6–101.2
Morphine sulfate	200 mcg/mL	D5W	C	99.9	99.4–100.3
Norepinephrine	64 mcg/mL	D5W	C	100.1	99.2–101.0
Paracetamol	10 mg/mL	U	C	100.0	99.4–100.6
Phenobarbitone	20 mg/mL	WFI	I ^b^	-	-
Piperacillin/tazobactam	200 mg/mL	WFI	C	101.6	101.0–102.2
Rifampicin	6 mg/mL	NS	I ^f^	-	-
Sodium bicarbonate	4.2% *w*/*v*	WFI	I ^b^	-	-
Vancomycin	10 mg/mL	D5W	C	100.4	99.4–101.4
Vecuronium	1 mg/mL	WFI	C	101.4	100.7–102.1
Parenteral nutrition PN 1	-	-	I ^a^	-	-
Parenteral nutrition PN 2	-	-	I ^a^	-	-
Parenteral nutrition PN 3	-	-	I ^a^	-	-
Parenteral nutrition PN 4	-	-	I ^a^	-	-
Parenteral nutrition PN 5	-	-	I ^a^	-	-
Parenteral nutrition PN 6	-	-	I ^a^	-	-

* P/C—Physicochemical compatibility; SIL—Sildenafil; C—Compatible; I—Incompatible; D5W—Glucose 5% *w/v*; WFI—Water for injection; NS—Normal saline/sodium chloride 0.9% *w/v*; U—Undiluted. ^a^—White precipitate appeared 5–10 min after mixing; ^b^—White precipitate appeared immediately after mixing; ^c^—Particles observed under polarized light; ^d^—Haze developed after mixing; ^e^—Milky turbidity appeared immediately after mixing; ^f^—Heavy precipitate appeared immediately after mixing—Colour could not be determined as the solution was coloured.

#### 3.2.2. Sildenafil 60 mcg/mL

Sildenafil 60 mcg/mL was physically compatible with all drug and PN fluid combinations except furosemide, meropenem and sodium bicarbonate (Table 2). The only combination shown to be physically compatible and chemically incompatible was ibuprofen. By contrast, sildenafil 60 mcg/mL was physically and chemically compatible with ibuprofen lysine.

**Table 2:** Physicochemical compatibility of secondary drugs and 2-in-1 parenteral nutrition solutions tested with sildenafil 60 mcg/mL, their concentrations and diluents.

Secondary Drug	Test Concentration	Diluent	P/C *	SIL * Ratio	95% CI of Ratio
Aciclovir	5 mg/mL	D5W	R	105.8	105.2–106.4
Amoxicillin	100 mg/mL	WFI	R	105.9	105.4–106.4
Amphotericin (Fungizone)	100 mcg/mL	D5W	R	104.2	102.8–105.7
Ampicillin	100 mg/mL	WFI	R	105.8	105.0–106.5
Flucloxacillin	50 mg/mL	D5W	R	105.7	104.9–106.5
Furosemide	1 mg/mL	D5W	I ^a^	-	-
Furosemide	0.2 mg/mL	D5W	I ^b^	-	-
Heparin	100 units/mL	NS	C	99.3	98.7–99.9
Hydrocortisone	10 mg/mL	NS	C	99.8	99.5–100.0
Hydrocortisone	1 mg/mL	NS	C	99.8	99.3–100.3
Ibuprofen	5 mg/mL	NS	I	**74.0**	72.9–75.1
Ibuprofen lysine	4 mg/mL	NS	C	99.4	98.9–99.9
Indometacin	200 mcg/mL	NS	C	99.1	98.7–99.5
Meropenem	50 mg/mL	NS	I ^b^	-	-
Meropenem	25 mg/mL	NS	I ^b^	-	-
Phenobarbitone	20 mg/mL	WFI	R	104.3	103.1–105.6
Rifampicin	6 mg/mL	NS	R	102.4	101.5–103.3
Sodium bicarbonate	4.2% *w*/*v*	WFI	I ^c^	-	-
Sodium bicarbonate	4.2% *w*/*v*	NS	I ^c^	-	-
Sodium bicarbonate	4.2% *w*/*v*	D5W	I ^c^	-	-
Parenteral nutrition PN 1	-	-	R	103.9	103.3–104.6
Parenteral nutrition PN 2	-	-	R	105.4	104.2–106.6
Parenteral nutrition PN 3	-	-	R	105.7	104.9–106.4
Parenteral nutrition PN 4	-	-	R	104.8	103.8–105.9
Parenteral nutrition PN 5	-	-	R	105.5	104.8–106.2
Parenteral nutrition PN 6	-	-	R	106.6	105.3–107.8

* P/C—Physicochemical compatibility; SIL—Sildenafil; C—Compatible; I—Incompatible; R—Re-test by filtration (see Table 3); D5W—Glucose 5% *w/v*; WFI—Water for injection; NS—Normal saline/sodium chloride 0.9% *w/v*; U—Undiluted. Bold SIL ratio shows chemical incompatibility. ^a^—White precipitate appeared 1 h after mixing; ^b^—Particles observed under polarized light; ^c^—Haze developed after mixing.

Thirteen drug combinations with sildenafil 60 mcg/mL, including the six PN solutions, resulted in sildenafil ratios >102% (Table 2). These combinations were re-tested, after filtering the combinations and control samples using nylon filters (Table 3). Apart from aciclovir and rifampicin (which were classified as compatible), all re-tested combinations of sildenafil with secondary drugs and PN solutions produced a significantly lower sildenafil ratio after filtration. The sildenafil ratio (filtered) was in the range of 90–110% for amoxicillin, ampicillin, phenobarbitone and three PN solutions; hence these combinations also were classified as compatible (Table 3). However, as the sildenafil ratio (filtered) was <90% for amphotericin, flucloxacillin and three PN solutions, possibly due to a sub-visible precipitate being filtered by the nylon filters (personal communication, C Locher and EKY Tang), these combinations were classified as incompatible (Table 3).

**Table 3:** Re-testing of drug combinations with sildenafil 60 mcg/mL in which SIL ratio (Table 2) was > 102%. Combinations considered compatible if sildenafil filtered ratio was in range of 90–110% (nylon filters; see methods for further details).

Secondary Drug	Test Concentration	SIL * Ratio (Unfiltered)	95% CI of Ratio (Unfiltered)	SIL * Ratio (Filtered)	95% CI of Ratio (Filtered)	P/C *
Aciclovir	5 mg/mL	107.1	106.3–108.0	106.1	104.2–108.0	C
Amoxicillin	100 mg/mL	105.9	103.6–108.1	98.3	95.4–101.3	C
Amphotericin (Fungizone)	100 mcg/mL	105.8	104.8–106.8	78.3	75.1–81.5	I
Ampicillin	100 mg/mL	102.6	100.0–105.2	94.4	92.2–96.5	C
Flucloxacillin	50 mg/mL	106.1	104.4–107.9	84.9	82.0–87.8	I
Phenobarbitone	20 mg/mL	102.8	100.8–104.8	95.5	92.7–98.3	C
Rifampicin	6 mg/mL	102.7	100.5–104.8	108.6	106.0–111.2	C
Parenteral nutrition PN 1	-	107.0	106.0–108.1	87.5	86.5–88.6	I
Parenteral nutrition PN 2	-	105.5	104.6–106.3	91.2	89.3–93.2	C
Parenteral nutrition PN 3	-	105.9	105.1–106.7	94.1	92.0–96.1	C
Parenteral nutrition PN 4	-	106.9	106.1–107.6	77.9	72.4–83.5	I
Parenteral nutrition PN 5	-	105.8	105.1–106.6	94.0	92.5–95.5	C
Parenteral nutrition PN 6	-	106.2	105.1–107.4	88.9	87.2–90.6	I

* SIL—Sildenafil; P/C—Physicochemical compatibility; C—Compatible; I—Incompatible.

### 3.3. Absorption/Adsorption Loss of Sildenafil by Filter Material 

The pilot study using 8 filters and 5 mL sildenafil solution showed the lowest drug recovery was in the first millilitre of the filtrate in all filters studied. For sildenafil 600 mcg/mL solution, the first millilitre had a drug recovery >90% in all filters tested (online Supplementary File Figure S7). In the second to fifth millilitres, drug recovery was >98%. However, for sildenafil 60 mcg/mL solution, only the nylon, polypropylene and inline ‘lipid’ filters showed a drug recovery of >90% in the first millilitre of the filtrate. All filter types showed a drug recovery >94% in the remainder of the sildenafil 60 mcg/mL filtrate (online Supplementary File Figure S8). 

**Figure 1:**
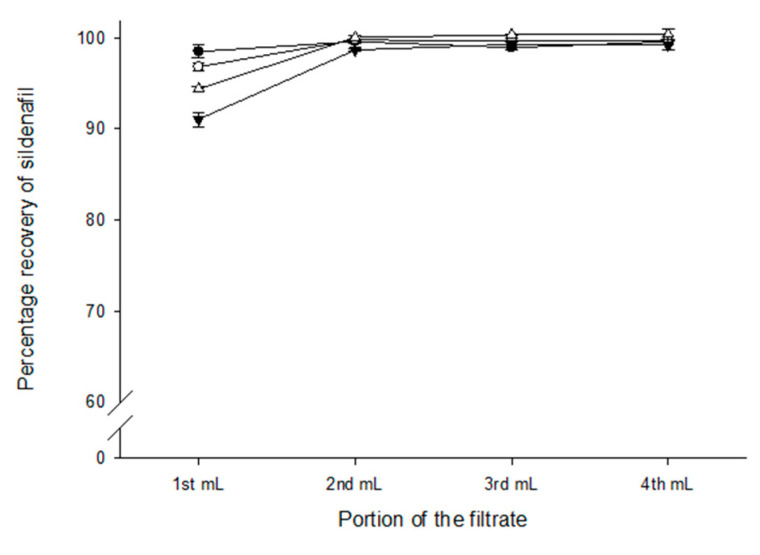
Recovery (%) of sildenafil 600 mcg/mL injection solution from sterilising filters. Sildenafil concentration was determined from each of four successive millilitres of solution passed through filters (• nylon; ○ polyethersulfone; ▼ mixed cellulose esters; △ inline polyethersulfone; see online Supplementary File Table S5 for further details). Data are mean ± SD (*n* = 3).

The filter test results obtained using the sildenafil commercial injection solution (600 mcg/mL) revealed that all filter types tested (NY, PES, MCE and Inline PES) showed a drug recovery >90% in the first millilitre of the filtrate (Figure 1). One way ANOVA results showed a statistically significant difference in drug recovery in the first millilitre compared to the remainder of the filtrate (*p* < 0.05). 

**Figure 2:**
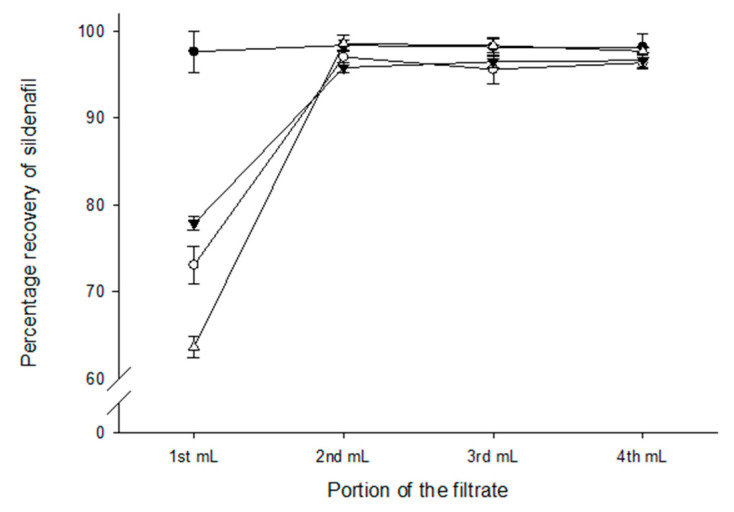
Recovery (%) of sildenafil 60 mcg/mL injection solution from sterilising filters. Sildenafil concentration was determined from each of four successive millilitres of solution passed through filters (• nylon; ○ polyethersulfone; ▼ mixed cellulose esters; △ inline polyethersulfone; see online Supplementary File Table S5 for further details). Data are mean ± SD (*n* = 3).

However, in the sildenafil 60 mcg/mL solution PES, MCE and inline filters showed a drug recovery <80% in the first millilitre of the filtrate (Figure 2). The drug recovery was >97% in all millilitre portions of the filtrate when the nylon filters were used and no statistically significant difference in drug recovery was observed between any millilitre portions. The first millilitre of the filtrate had a statistically significantly lower drug recovery (*p* < 0.05) than the remaining filtrate in all other filters used.

## 4. Discussion

Our study has demonstrated that sildenafil 600 mcg/mL injection was physically and chemically compatible with 29 IV drugs at high-end, clinically relevant concentrations for NICU settings (Table 1). None of these drugs were tested at lower concentrations or against sildenafil 60 mcg/mL in the present study. Rather, it was concluded that lower drug concentrations would also be compatible.

Sixteen of the secondary drugs (at their standard or high-end clinically relevant concentration), and all six 2-in-1 PN solutions, were physically incompatible with sildenafil 600 mcg/mL (Table 1). Nine of these sixteen drugs were evaluated at only one relevant concentration and subsequently tested against sildenafil 60 mcg/mL. A further four were evaluated at lower, clinically relevant concentrations and found to be physically incompatible (amoxicillin, ampicillin, furosemide and meropenem); hence, sildenafil 600 mcg/mL was deemed incompatible with 13 of the 45 IV drugs at concentrations relevant to NICU settings. However, sildenafil 600 mcg/mL was found to be compatible with three drugs at low concentrations (calcium gluconate 50 mg/mL, heparin 2 units/mL and hydrocortisone 1 mg/mL; Table 1), which could be co-administered at these lower, clinically relevant concentrations if required. The results for heparin align with previous data indicating that heparin was incompatible at higher concentration (100 units/mL) [8] and compatible at a lower concentration (1 unit/mL) [9]. Furthermore, the calcium gluconate concentration used for urgent correction of hypocalcaemia is 50 mg/mL [13] and this concentration was found to be physicochemically compatible with sildenafil 600 mcg/mL. Hence, calcium gluconate was not tested with sildenafil 60 mcg/mL.

Fifteen drugs and the six 2-in-1 PN solutions were tested against the lower sildenafil concentration of 60 mcg/mL, which is used in preterm infants [3]. Four drugs showed physical and chemical compatibility, three were physically incompatible and one (ibuprofen) was chemically incompatible (Table 2). The remaining seven drugs and the PN solutions were found to have sildenafil ratios >102%. Although there was no visible or microscopic evidence of precipitation (including Tyndall beam and magnified polarised light observation), we were aware of unpublished data suggesting sub-visible precipitates for other drug combinations (personal communication, C Locher and EKY Tang). Therefore, a series of filter validation studies were conducted which identified 0.2 µm nylon filters as the most suitable, and these combinations were investigated before and after filtering (Table 3). Based on pre-determined criteria for the 90–110% sildenafil ratio (filtered), it was concluded that aciclovir, amoxicillin, ampicillin, phenobarbitone and rifampicin were compatible with sildenafil 60 mcg/mL, but amphotericin and flucloxacillin were incompatible. Three of the PN solutions were also classified as compatible, however, there were no notable features of these three formulations (#2, #3 and #5) compared to the incompatible formulations and further investigation of this finding was beyond the scope of the present study.

Physical incompatibilities in the present study ranged from florid precipitation to hazy fluids and potential sub-visible precipitation. The former were generally visible to the naked eye, where the limit of detection is approximately 100 µm for discrete particles and 10 µm for hazy or cloudy fluids [21], the observation of which may be enhanced by polarised light [10] or Tyndall beam [22]. Sub-visible particles in the order of 1–2 µm also may be detected by the visual enhancement techniques or light microscopy, however, it has been postulated that incompatible drug combinations could cause nano- or micro-precipitation, ostensibly <1 µm (personal communication, C Locher and EKY Tang). In the present study, sub-detectable precipitation may explain the substantially lower sildenafil ratio after 0.2 µm filtration for amphotericin, flucloxacillin and three PN solutions. Although the clinical impact of injection of particulate matter <1 µm is unclear, the pre-determined criteria for the sildenafil ratio (outside the range of 90–110%) was applied in the present study to define incompatible drug combinations and recommend avoidance in NICU clinical settings.

The present study included some potential limitations that are consistent with previous investigations of physicochemical compatibility. For example, due to the resource constraints and unclear interpretation or clinical significance of pH changes [23], the determination of pH was not performed (the volume of drug solutions required for pH determination would be >5 mL, and placing a wet pH probe into consecutive samples would reduce the drug concentration and may produce false results). As pH changes may contribute to chemical reaction [24] or altered drug solubility [25], the use of HPLC analysis in the present physicochemical study would likely counter the need for pH analysis. Another potential issue was conducting HPLC analysis only for the primary drug (sildenafil). This is consistent with previous IV physicochemical compatibility studies where a large number of secondary drugs have been tested [26,27,28,29]. However, there are some reports where both the primary and secondary drugs have been assayed, typically in studies where a modest range of secondary drugs have been tested [8,16,18]. HPLC analysis of both the primary and secondary IV drugs would have significant cost and complexity implications, to ensure validated HPLC assays were developed for each secondary drug. Consequently, we assumed that physicochemical incompatibility would cause a decline in the concentration of both IV drugs and be detected by HPLC assay of the primary drug. Nevertheless, there may be situations where quantifying the secondary drug concentration is of potential value if chemical incompatibility is suspected or inconclusive results require further investigation.

A potential limitation related to clinical interpretation of the present study was the drug combination contact time of 2 h, which was based on a previous report that 60 min was a plausible maximum contact time for two drug solutions in the IV tubing from the Y-site to the tip of a cannula in NICU settings [30]. By comparison, a four hour study duration is commonly used for drug compatibility studies and may be applicable to other clinical settings [14,15,16,17,31,32,33,34,35]. A further clinical consideration is that the present study and most IV compatibility research has been conducted at room temperature [31,32,34,35,36], which is comparable to the ambient temperature in the majority of clinical settings, including NICU. However, whilst the IV drugs in syringes (or other delivery devices) and a proportion of the IV tubing in NICU will most likely be at room temperature, part of the IV tubing may be inside a humidicrib at up to 37 °C and some recent IV compatibility studies have been conducted at elevated temperature to simulate the humidicrib environment [8,37].

## 5. Conclusions

Sildenafil 600 mcg/mL was physicochemically compatible with approximately 70% of the 45 clinically relevant IV drugs used in NICU settings that were tested in the present study. A further seven drugs were compatible with sildenafil 60 mcg/mL. Six drugs (amphotericin, flucloxacillin, furosemide, ibuprofen, meropenem and sodium bicarbonate) were incompatible with sildenafil and should not be co-administered via Y-site infusions. Six 2-in-1 PN solutions were incompatible with sildenafil 600 mcg/mL; however, three appeared to be compatible with sildenafil 60 mcg/mL and three were deemed incompatible. Sildenafil solution was compatible with nylon syringe filters; however, absorption/adsorption loss from the first millilitre of filtrate occurred with polyethersulfone and cellulose ester filters, which should be avoided for small volumes and/or low concentrations of sildenafil solution. 

## Data Availability

Data not provided in the manuscript are available upon reasonable request to the authors.

## Supplementary Materials

The following supporting information can be downloaded at: https://www.mdpi.com/article/10.3390/pharmaceutics16030419/s1, Figure S1: Sildenafil 600 mcg/mL exposure to 20% *v*/*v* hydrogen peroxide; Figure S2: Sildenafil 600 mcg/mL exposure to 4 M NaOH; Figure S3: Sildenafil 600 mcg/mL exposure to 4 M HCl; Figure S4: Sildenafil 600 mcg/mL in water (1:1 *v*/*v*) exposure to heat; Figure S5: Sildenafil 600 mcg/mL in water (1:1 *v*/*v*) exposure to laboratory fluorescent lighting 24/7 and normal daylight; Figure S6: Linearity curve for sildenafil solution in aqueous solution within the concentration range 3–800 mcg/mL (*n* = 3); Figure S7: Sildenafil percentage recovery by different filters using the 600 mcg/mL solution; Figure S8: Sildenafil percentage recovery by different filters using the 60 mcg/mL solution; Figure S9: Photograph and corresponding photomicrograph of physical incompatibility (white precipitate) of sildenafil 600 mcg/mL with furosemide 1 mg/mL; Figure S10: Photograph and corresponding photomicrograph of physical incompatibility (haze) of sildenafil 600 mcg/mL with heparin 100 units/mL; Figure S11: Photograph and corresponding photomicrograph of physical incompatibility (precipitate in an originally coloured solution) of sildenafil 600 mcg/mL with rifampicin 6 mg/mL; Figure S12: Photograph and corresponding photomicrograph of physical incompatibility (particles under polarized light) of sildenafil 600 mcg/mL with calcium gluconate 100 mg/mL Table S1: Manufacturers/suppliers of injectable products used for compatibility studies; Table S2: Composition of the 2-in-1 parenteral nutrition solutions, manufactured at King Edward Memorial Hospital; Table S3: Accuracy, intra-assay and inter-assay precision data for selected sildenafil concentrations; Table S4: Robustness test results for deliberate changes in method parameters; Table S5: Syringe filter types tested, the membrane and mesh size description.
