# Supplementary material for: The Physicochemical Compatibility of Sildenafil Injection with Parenteral Medications Used in Neonatal Intensive Care Settings
Source: Pharmaceutics. 2024 Mar 18;16(3):419. doi: 10.3390/pharmaceutics16030419 (PMC10974670; doi:10.3390/pharmaceutics16030419)
Supplement: Supplementary file 1 [file pharmaceutics-16-00419-s001.zip › pharmaceutics-2863917-supplementary.pdf]

Supplementary information

Physicochemical compatibility of sildenafil injection with parenteral medications used in neonatal intensive care settings

D. Thisuri N. De Silva,[1] Tobias Strunk,[2,3,4] Michael Petrovski,[5] Madhu Page-Sharp,[1] Brioni R. Moore,[1,2,3,6] and Kevin T. Batty[1,6*]

[1] Curtin Medical School, Curtin University, Bentley, Western Australia

[2] Medical School, The University of Western Australia, Crawley, Western Australia

[3] Wesfarmers Centre for Vaccines and Infectious Diseases, Telethon Kids Institute, Nedlands, Western Australia

[4] Neonatal Directorate, King Edward Memorial Hospital, Child and Adolescent Health Service, Subiaco, Western Australia

[5] Pharmacy Department, King Edward Memorial Hospital, Women and Newborn Health Service, Subiaco, Western Australia

[6] Curtin Health Innovation Research Institute, Curtin University, Bentley, Western Australia

* Corresponding author: Professor Kevin Batty, Curtin Medical School, Curtin University, GPO Box U1987, Perth, Western Australia, 6845. Telephone: (61-8) 9266 2535; Email: Kevin.Batty@curtin.edu.au

Section 1: Manufacturers of the injectable products used for compatibility studies (Table S1) and composition of the 2-in-1 parenteral nutrition solutions (Table S2)

Table S1. Manufacturers/ suppliers of injectable products used for compatibility studies

**Injectable drug**	**Manufacturer/ supplier**	**Lot No**
Aciclovir	Pfizer Australia Pty Ltd, Sydney, NSW, Australia	FT1848AA
Alprostadil	Pfizer Australia Pty Ltd, Sydney, NSW, Australia	FD6386
Amoxicillin	Juno Pharmaceuticals Pty Ltd, Cremorne, VIC, Australia	IC02KH
Amphotericin B - Fungizone	Neon Healthcare Ltd, Mill Studio Business Centre, Ware, UK	28631TB21
Amphotericin B - Liposomal	Gilead Sciences Pty Ltd, St Kilda Road, Melbourne, VIC, Australia	028780
Ampicillin	Juno Pharmaceuticals Pty Ltd, Cremorne, VIC, Australia	0P03AY
Benzylpenicillin	Seqirus (Australia) Pty Ltd, Melbourne, VIC, Australia	KT4974
Caffeine base*	Perth Children's Hospital, Nedlands, WA, Australia	6018
Caffeine citrate	Phebra Pty Ltd, Lane Cove West, NSW, Australia	14806
Calcium gluconate	Phebra Pty Ltd, Lane Cove West, NSW, Australia	14688
Cefotaxime	Pfizer Australia Pty Ltd, Sydney, NSW, Australia	G105HA0
Ciprofloxacin	Aspen Pharmacare Australia Pty Ltd, St Leonards, NSW, Australia	10597
Clonidine	Medicianz Healthcare Pty Ltd, Melbourne, VIC, Australia	CLA032
Cloxacillin	Xion Pharmaceuticals Pty Ltd, Pune, India	101
Dexmedetomidine	Accord Healthcare Pty Ltd, Melbourne, VIC, Australia	M2110403
Dobutamine	Pfizer Australia Pty Ltd, Sydney, NSW, Australia	FY3034AA
Dopamine	Juno Pharmaceuticals Pty Ltd, Cremorne, VIC, Australia	A21F3R
Epinephrine	Aspen Pharmacare Australia Pty Ltd, St Leonards, NSW, Australia	AS116A1
Fentanyl citrate	Piramal Critical Care Pty Ltd, Chatswood, NSW, Australia	HC5M
Flucloxacillin	Juno Pharmaceuticals Pty Ltd, Cremorne, VIC, Australia	0X18HY
Fluconazole	Pfizer Australia Pty Ltd, Sydney, NSW, Australia	B560704
Furosemide	Baxter Health care Pty Ltd, Old Toongabbie, NSW, Australia	B5E0004A
Gentamicin	Pfizer Australia Pty Ltd, Sydney, NSW, Australia	B785
Heparin	Pfizer Australia Pty Ltd, Sydney, NSW, Australia	159049A
Hydrocortisone	Pfizer Australia Pty Ltd, Sydney, NSW, Australia	ER8089
Ibuprofen	Seqirus (Australia) Pty Ltd, Melbourne, VIC, Australia	10098R
Ibuprofen lysine	Prasco Laboratories, Commerce Ct, Mason, United States	B215329
Indometacin	Promedica SRL, Via Palermo, Parma, Italy	22906
Insulin (Actrapid®)	Novo Nordisk Pharmaceuticals Pty Ltd, Baulkham Hills, NSW, Australia	LR79K53
Levetiracetam	Apotex Pty Ltd, Macquarie Park, NSW, Australia	275447
Linezolid	Fresenius Kabi Australia Pty Ltd, Mount Kuring-gai, NSW, Australia	15PDE270
Meropenem	Sun Pharma ANZ Pty Ltd, Macquarie Park, NSW, Australia	DFC2162A
Metronidazole	Juno Pharmaceuticals Pty Ltd, Cremorne, VIC, Australia	10221
Midazolam	Pharmaco (Australia) Ltd, Gordon, NSW, Australia	F0021F01
Milrinone	Sanofi-Aventis Australia Pty Ltd, Macquarie Park, NSW, Australia	J0496
Morphine hydrochloride	Juno Pharmaceuticals Pty Ltd, Cremorne, VIC, Australia	A22A18
Morphine sulfate	Pfizer Australia Pty Ltd, Sydney, NSW, Australia	212004
Norepinephrine	Juno Pharmaceuticals Pty Ltd, Cremorne, VIC, Australia	208599
Paracetamol	B.Braun Australia Pty Ltd, Bella Vista, NSW, Australia	21436451

Injectable drug	Manufacturer/ supplier	Lot No
Phenobarbitone	Aspen Pharmacare Australia Pty Ltd, St Leonards, NSW, Australia	042344
Piperacillin/tazobactam	Sandoz Pty Ltd, Macquarie Park, NSW, Australia	LN1377
Rifampicin	Sanofi-Aventis Australia Pty Ltd, Macquarie Park, NSW, Australia	0J6511
Sildenafil	Viatris Australia Pty Ltd, Sydney, NSW, Australia	B710506
Sodium bicarbonate	Phebra Pty Ltd, Lane Cove West, NSW, Australia	14924a
Vancomycin	Hospira Australia Pty Ltd, Mulgrave, VIC, Australia	J016913AA
Vecuronium	Sun Pharma ANZ Pty Ltd, Macquarie Park, NSW, Australia	HAC2372A

*Caffeine base 10 mg/mL injection comprises caffeine, sodium chloride, hydrochloride acid and Water for Injection; the injection is isotonic and has a pH approximately 4.2 (AUSPMAN/ Perth Children's Hospital)

Table S2. Composition of the 2-in-1 parenteral nutrition solutions, manufactured at King Edward Memorial Hospital

	PN 1<br>Preterm A	PN 2<br>Preterm B	PN 3<br>Term	PN 4<br>Custom 1	PN 5<br>Custom 2	PN 6<br>Custom 3
Amino acids (Primine), g/100mL	2.98	2.98	2.3	0.5	3.5	2.3
Glucose, g/100mL	4.96	7.94	12	2	14	8
Sodium, mmol/100mL	3.97	3.97	4	4	4	4
Potassium, mmol/100mL	1.99	1.99	2	2	2	2
Calcium, mmol/100mL	1.49	1.49	0.9	1.5	1.5	1.5
Magnesium, mmol/100mL	0.25	0.25	0.25	0.25	0.25	0.25
Phosphate, mmol/100mL	1.49	1.49	0.9	0.5	1.5	1.5
Chloride, mmol/100mL	2	2	2.54	1.8	2.08	1.97
Acetate, mmol/100mL	1.99	1.99	2.6	1.79	2.08	1.96
Heparin, units/100mL	49.63	49.63	50	50	50	50
Trace elements, mL/100mL	0.73	0.73	0.74	0.74	0.74	0.74

Section 2. Method validation - chromatograms of sildenafil degradation experiments (Figure S1 – 5), sildenafil linearity curve (Figure S6), accuracy, precision (Table S3) and robustness testing results of HPLC method (Table S4)

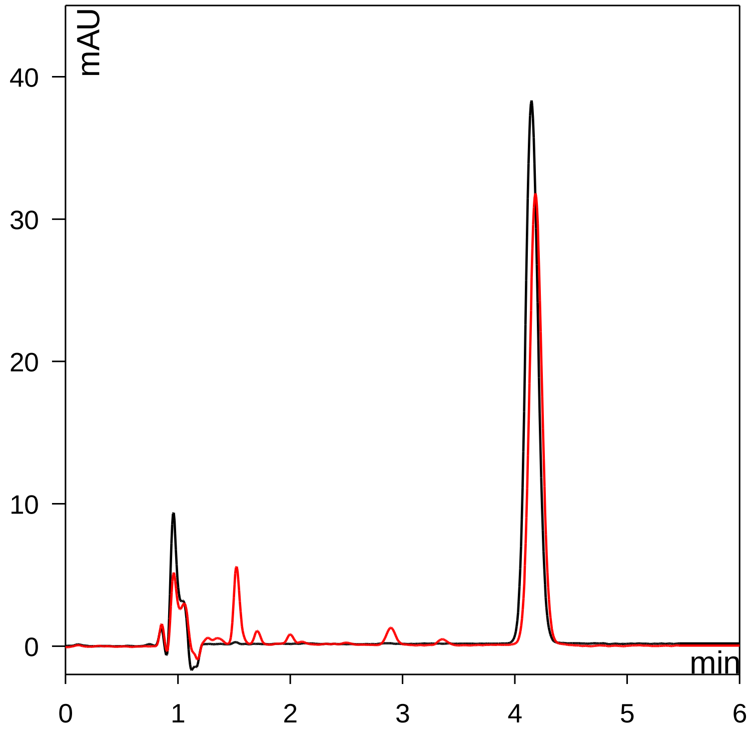

Figure S1 – Sildenafil 600 mcg/mL exposure to 20% v/v hydrogen peroxide (1:1 v/v), stored at 45°C; Sample diluted 1-in-50 with water at the point of assay; injection volume 20 µL. (– t0; – Day 7); Degradation products were detected at 1.5, 1.7 and 2.9 min, at day 7

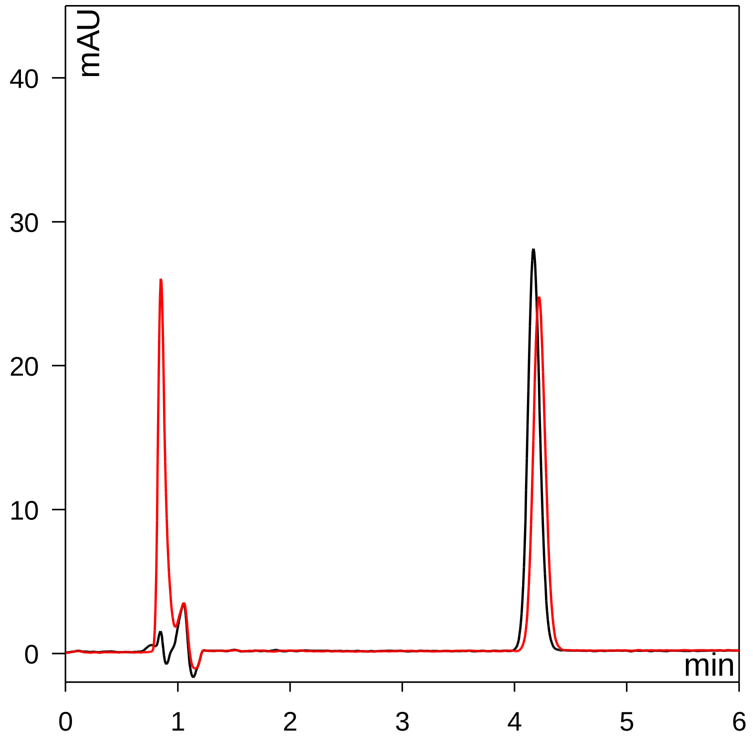

Figure S2 – Sildenafil 600 mcg/mL exposure to 4 M NaOH (1:1 v/v), stored at 45°C; Sample neutralized with 4 M HCl and diluted 1-in-50 with water at the point of assay; Injection volume 20 µL. (− t0; − Day 7); A degradation product was detected 0.9 min, at day 7

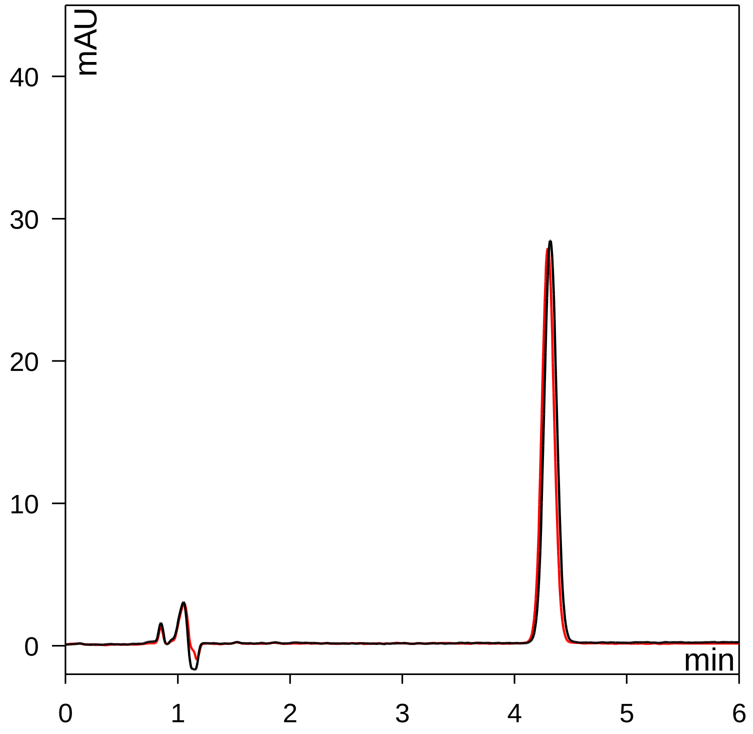

Figure S3 – Sildenafil 600 mcg/mL exposure to 4 M HCl (1:1 v/v), stored at 45°C; Sample neutralized with 4 M NaOH and diluted 1-in-50 with water at the point of assay; Injection volume 20 µL. (– t0; – Day 7); No degradation products observed

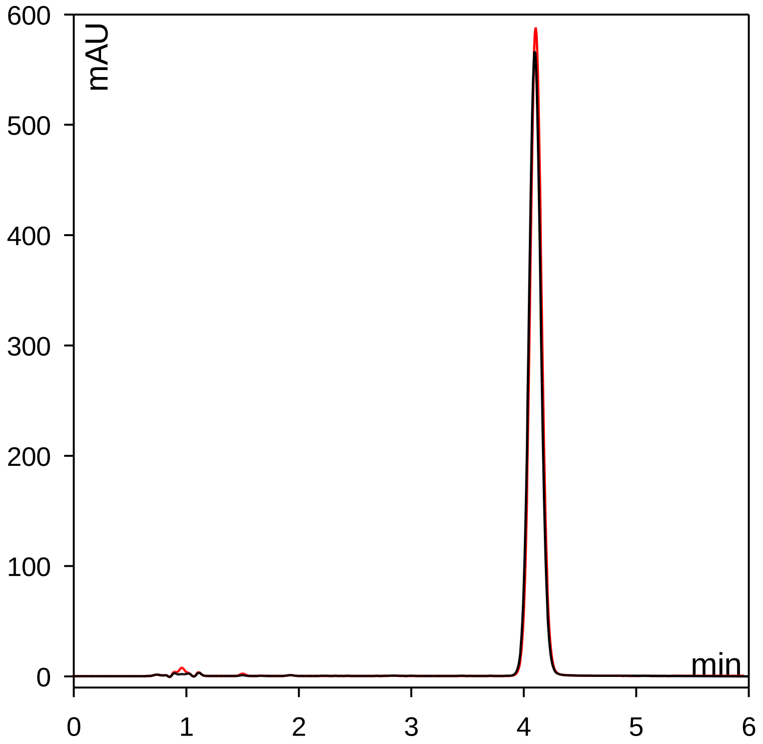

Figure S4 – Sildenafil 600 mcg/mL in water (1:1 v/v) exposure to heat at a temperature of 60°C in a water bath; Injection volume 5 µL (– t0; – Day 3); No degradation products observed

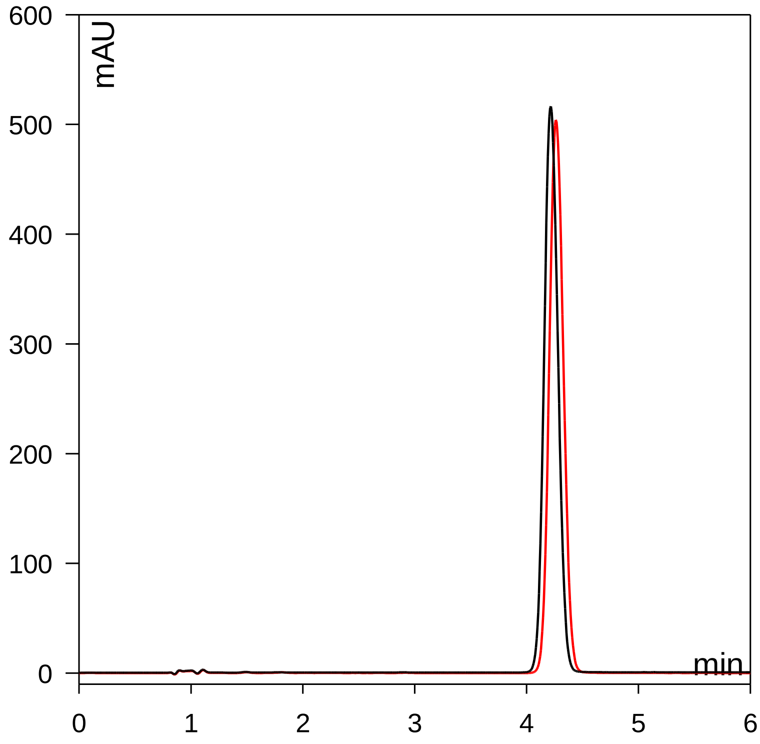

Figure S5 – Sildenafil 600 mcg/mL in water (1:1 v/v) exposure to laboratory fluorescent lighting 24/7 and normal daylight (indirect sunlight) for approximately 12 h per day at 22°C (room temperature); Injection volume 5 µL; (– t0; – Day 7); No degradation products observed

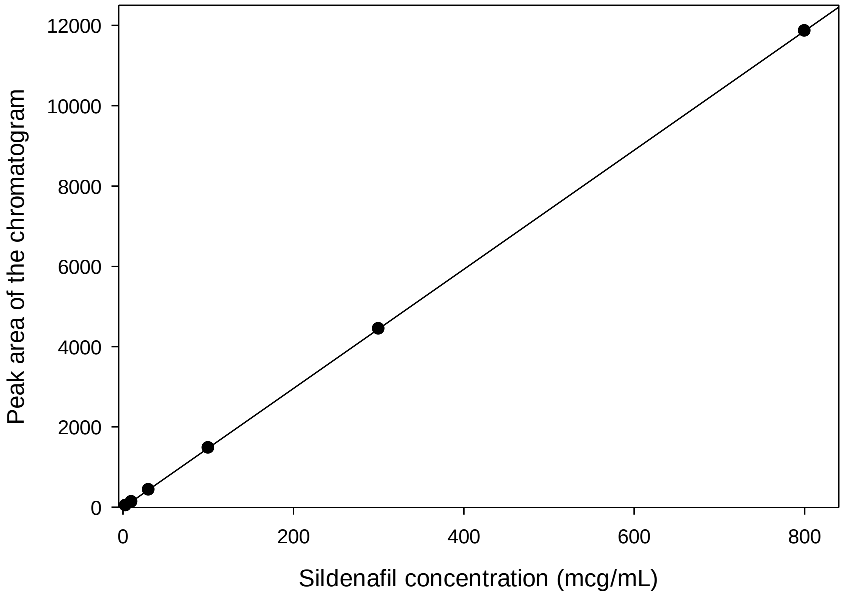

Figure S6 - Linearity curve for sildenafil solution in aqueous solution within the concentration range 3 – 800 mcg/mL (n = 3); Correlation coefficient (r^2) >0.999; Regression equation Y = 14.86x – 10.036

Accuracy and precision of the developed HPLC method

For accuracy testing, separate sildenafil solution series was prepared with concentrations of 600, 100, 10 and 2.9 (LLOQ) mcg/mL (n = 5) using the sildenafil reference standard and the commercial sildenafil injection diluted with D5W. The chromatogram peak areas of the two series were compared, and accuracy was expressed as a percentage of the reference standard solution's peak area (nominal concentration), for the particular concentration level (Table S3).

Intra-assay precision was calculated using the percentage standard deviation (%RSD) of the peak areas of the five replicates of each concentration level of sildenafil injection solutions, in a single assay (Table S3).

Inter-assay precision was determined by calculating the %RSD pooled, for the same sildenafil concentration levels of sildenafil injection solutions, in three assays analysed on different days (Table S3).

Table S3: Accuracy, intra-assay and inter-assay precision data for selected sildenafil concentrations

Sildenafil concentration (mcg/mL); n = 5	Sildenafil concentration as a % of nominal concentration (Mean ± SD, n=5)	Intra assay-precision (% RSD)	Inter-assay precision (% RSD pooled)
LLOQ	100.2 ± 2.5	2.2	4.2
10	104.0 ± 0.8	3.1	2.0
100	104.6 ± 0.5	0.7	0.6
600	104.0 ± 0.4	0.3	0.3

Robustness of the developed HPLC method

The robustness of an analytical procedure is a measure of its capacity to remain unaffected by small, but deliberate variations in method parameters and provides an indication of its reliability during normal usage. To evaluate robustness, sildenafil 100 mcg/mL samples (from sildenafil standard and commercial injection; n = 5) were tested using the modified method. Changes with respect to standard method parameters included flow rate (0.8 mL/min) and mobile phase composition (Acetonitrile: Buffer ratio 45:55). The accuracy of the modified methods was compared with the standard method. The percentage concentrations of sildenafil in robustness testing experiment revealed that the method was robust despite deliberate minute changes in method parameters (Table S4).

Table S4: Robustness test results for deliberate changes in method parameters

Parameters	Conditions	Sildenafil concentration as a % of nominal concentration (Mean ± SD, n=5)
Flow rate	1 mL/min	105.1 ± 1.0
	0.8 mL/min	105.1 ± 1.1
Mobile phase composition (Acetonitrile: Buffer)	40:60	105.1 ± 1.0
	45:55	105.2 ± 1.0

System suitability

A potential limitation of the study is the exclusion of peak purity analysis. However, to confirm system suitability, a sildenafil reference standard solution of 300 mcg/mL (the nominal sildenafil concentration of sildenafil and secondary drug combination samples) was analysed using the HPLC method alongside the compatibility samples in each assay. The %RSD of the retention time, peak area and the peak symmetry of sildenafil peak were 1.6, 2.0 and 2.3 respectively. The peak symmetry for sildenafil remained between 0.9 – 1.04, throughout the experimenting period.

Section 3. Sildenafil filter testing data – Syringe filter types tested (Table S5), Sildenafil percentage recovery by different filters – pilot study (Figure S7 – 8)

Table S5: Syringe filter types tested, the membrane and mesh size description

Abbreviation	Description	Manufacturer/ Supplier
RC	Regenerated Cellulose, 15 mm diameter, 0.2 µm membrane, non-sterile	Phenomenex Australia Pty Ltd, 2 Chaplin Dr, Lane Cove West NSW 2066
NY	Nylon, 15 mm diameter, 0.2 µm membrane, non-sterile	Phenomenex Australia Pty Ltd, 2 Chaplin Dr, Lane Cove West NSW 2066
PVDF	Polyvinylidene Fluoride, 15 mm diameter, 0.2 µm membrane, non-sterile	Phenomenex Australia Pty Ltd, 2 Chaplin Dr, Lane Cove West NSW 2066
PES	Millex-GP, Polyethersulfone, 33 mm, 0.22 µm membrane, sterile	Merck Millipore Ltd., Tullagreen, Carrigtwohill, County Cork, Ireland
MCE	Millex-GS, Mixed cellulose esters, 33 mm, 0.22 µm membrane, sterile	Merck Millipore Ltd., Tullagreen, Carrigtwohill, County Cork, Ireland
GHP	Hydrophilic polypropylene 13 mm, 0.2 µm membrane, non-sterile	Pall Life Sciences 1 - 2 Wandarri Court, Cheltenham 3192, Melbourne, Australia
Inline	Polyethersulfone, 0.2 µm membrane, sterile	Pall Medical, Avenue de Tivoli 3, CH-1700 Fribourg, Switzerland
Inline lipid	Lipid filter, 1.2 µm membrane, sterile	Codan US Corporation, USA

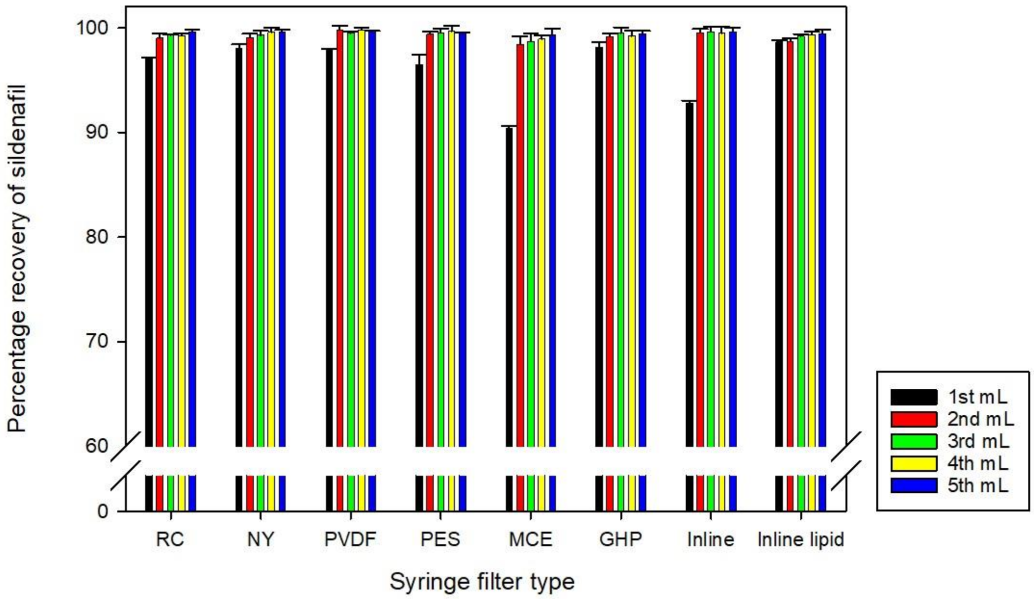

Figure S7- Sildenafil percentage recovery by different filters using the 600 mcg/mL solution; The coloured bars represent the recovery in five separate, consecutive millilitre portions – pilot study

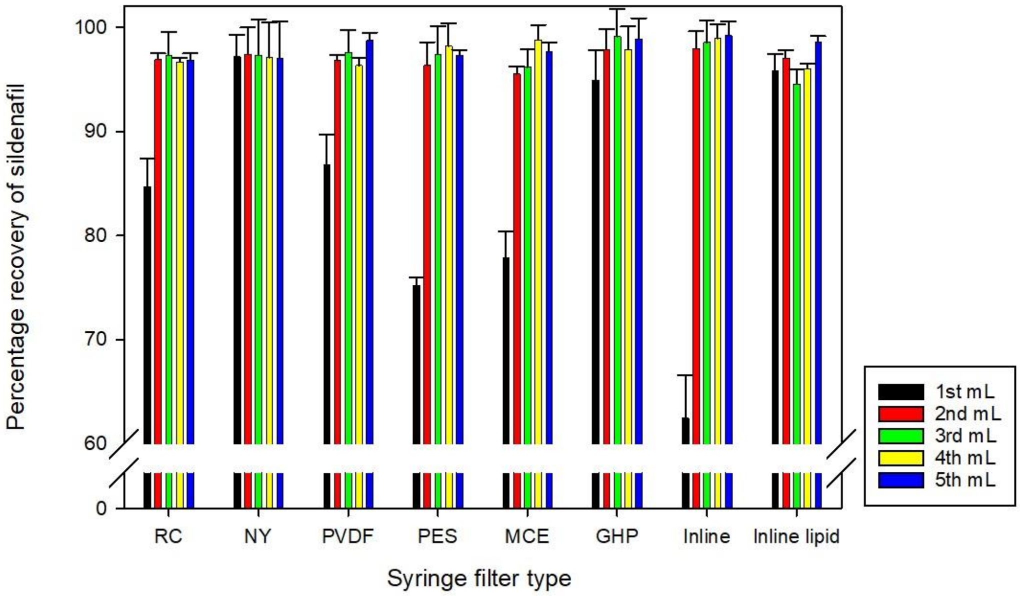

Figure S8 - Sildenafil percentage recovery by different filters using the 60 mcg/mL solution; The coloured bars represent the recovery in five separate, consecutive millilitre portions – pilot study

Section 4. Photographs and corresponding photomicrographs of physical incompatibilities of sildenafil 600 mcg/mL with frusemide, heparin, rifampicin, and calcium gluconate (Figure S9 – 12)

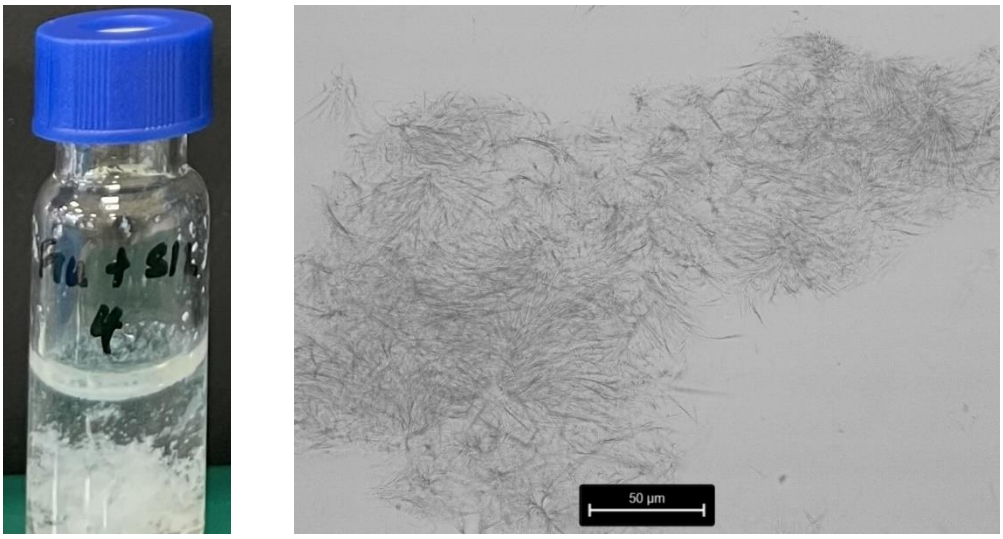

Figure S9 - Photograph (left) and corresponding photomicrograph (right) (Leica MC190HD, objective x40) of physical incompatibility (white precipitate) of sildenafil 600 mcg/mL with furosemide 1 mg/mL

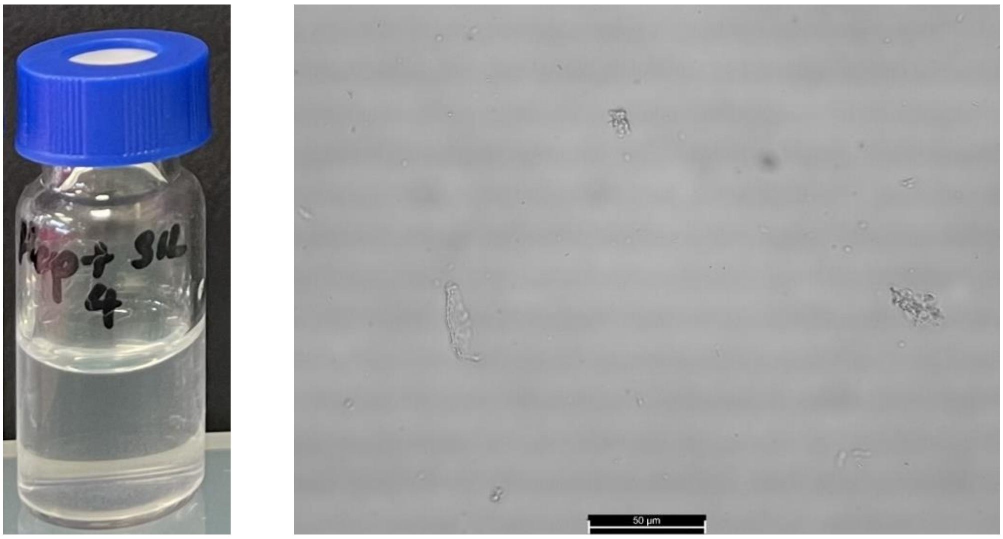

Figure S10 - Photograph (left) and corresponding photomicrograph (right) (Leica MC190HD, objective x40) of physical incompatibility (haze) of sildenafil 600 mcg/mL with heparin 100 units/mL

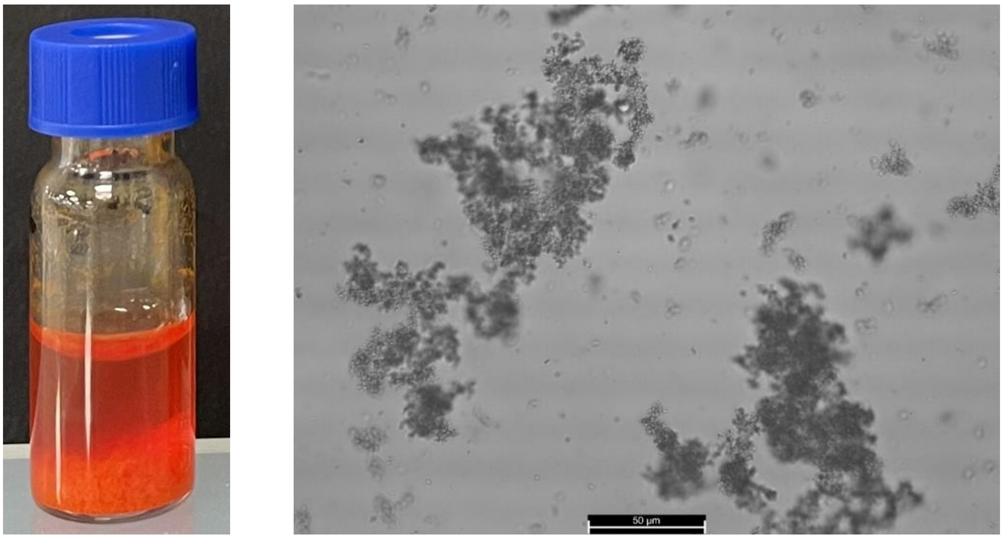

Figure S11 - Photograph (left) and corresponding photomicrograph (right) (Leica MC190HD, objective x40) of physical incompatibility (precipitate in an originally coloured solution) of sildenafil 600 mcg/mL with rifampicin 6 mg/mL

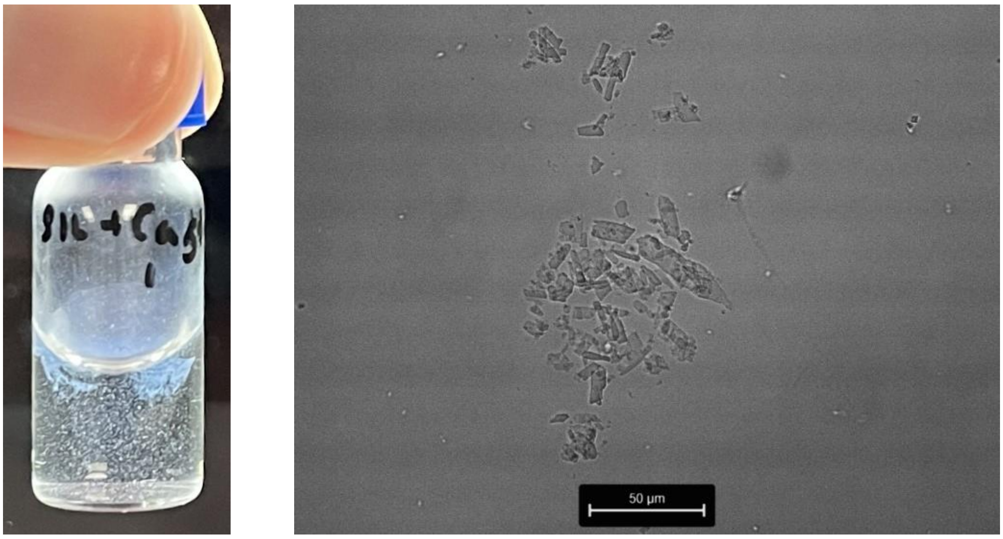

Figure S12 - Photograph (left) and corresponding photomicrograph (right) (Leica MC190HD, objective x40) of physical incompatibility (particles under polarized light) of sildenafil 600 mcg/mL with calcium gluconate 100 mg/mL
